# Mental illness stigma among perinatal women in low- and middle-income countries: early career psychiatrists' perspective

**DOI:** 10.3389/fpsyt.2023.1283715

**Published:** 2023-12-05

**Authors:** Arpana Pokharel, Sharad Philip, Murchana Khound, Samer El Hayek, Renato de Filippis, Ramdas Ransing, Mohsen Heidari Mokarar, Maryam Orooji, Mohammadreza Shalbafan

**Affiliations:** ^1^Department of Psychiatry, Clinical Neurosciences, and Addiction Medicine, All India Institute of Medical Sciences, Guwahati, India; ^2^Department of Psychiatry, Devdaha Medical College, Butwal, Nepal; ^3^Department of Pediatrics, All India Institute of Medical Sciences, Guwahati, India; ^4^Medical Department, Erada Center for Treatment and Rehabilitation in Dubai, Dubai, United Arab Emirates; ^5^Psychiatry Unit, Department of Health Sciences, University Magna Graecia of Catanzaro, Catanzaro, Italy; ^6^Department of Psychiatry, Faculty of Medicine, Prince of Songkla University, Hat Yai, Thailand; ^7^Department of Psychiatry, Imam Hossein Hospital, School of Medicine, Alborz University of Medical Sciences, Karaj, Iran; ^8^Mental Health Research Center, Psychosocial Health Research Institute, Department of Psychiatry, School of Medicine, Iran University of Medical Sciences, Tehran, Iran

**Keywords:** mental health, mental disorders, psychiatry, perinatal care, social stigma

## Introduction

Perinatal mental disorders (PMDs) are prevalent among women in low- and middle-income countries (LMICs), constituting the most frequently encountered complications during the perinatal period, affecting almost 20% of perinatal women (1). The PMDs are associated with adverse obstetrics outcomes (e.g., increased risk of pre-eclampsia, antepartum and postpartum hemorrhage, placental abruption and still-births) and neonatal outcome (e.g., preterm births and fetal growth impairments) (2). PMDs are among the commonest morbidities of pregnancy, associated with high rates of maternal mortality and adverse outcomes, however, these conditions are often remains underdiagnosed and undertreated in LMICs. Lack of access to perinatal mental health services is mainly due to a lack of adequate resources (human, financial), inadequate knowledge among health care workers, stigma, a negative attitude toward mental disorders, a lack of evidence-based integrative or collaborative models of care, and being overlooked by stakeholders (3, 4). Mental illness stigma is also a major barrier to help-seeking, and it contributes to poor quality of life and social withdrawal among person with mental illness. Addressing the stigma associated with perinatal mental disorders has the potential to improve perinatal mental care inclusive of screening, and intervention and thus preventing the complications. However, stigma among perinatal women remains largely unexplored in LMICs (5). In this article, we-a team of early-career psychiatrists (ECPs) attempted to explore stigma toward mental illness among perinatal women residing in LMICs, its impact, and interventions to reduce it using a theoretical model. Table 1 summarizes the stigma associated with perinatal mental illness, its types, possible impact, and interventions. The theme-based approach was used to describe the types of stigma among perinatal women and to propose interventions (8).

**Table 1:** Stigma toward perinatal mental illness: types, potential impact, and interventions.

**Type of stigma**	**Potential impact**	**Potential interventions**
Public stigma/experienced stigma: Perinatal women face discrimination or prejudice as a result of their mental illness. The negative attitude of medical professionals toward taking any psychiatric medications during pregnancy (6).	• Social isolation and avoidance, lack of help-seeking behavior. • Increase in psychiatric morbidities. • Decrease self-esteem, quality of life, and self-efficacy.	• Peer Educator intervention. • Providing social support. • Role play • Lecture-based education. • Brief training or longer training intervention. • Training sessions through the media (Anti-stigma movies) (7)
Self-stigma/internalized stigma: Mothers with mental illnesses apply negative/judgmental attitudes displayed by others to themselves (8, 9).		
Associated stigma: Family members of a perinatal woman with a mental illness are stigmatized for being associated with her.		
Structural stigma: Perinatal women with mental illness are denied from infant care and community participation.		
Label avoidance: Perinatal women may avoid seeking mental health care as it will automatically label them as having a mental illness.		

## Impact of stigma toward perinatal mental illness on mother and baby

Few studies attempted to investigate stigma toward perinatal mental illness in LMICs (9, 10). In our experience, perinatal women with mental illness are frequently stigmatized for seeking treatment, refusing treatment, being on medications, and being associated with a husband or family member (e.g., mother) with a mental illness in LMICs. This frequently results in social withdrawal of perinatal women with mental health conditions, financial burden on the family, and family disruption (e.g., divorce). These negative consequences exacerbate mental health issues and perpetuate the stigmatization cycle. Stigma leads to non-treatment of common perinatal mental disorders. This increases the risk of suicide and substance use among perinatal women and is linked to poor infant outcomes (e.g., preterm delivery, developmental and cognitive delays, and attachment and bonding problems between mother and child) (9, 11, 12) (Table 1). Furthermore, secondary to stigma, it becomes difficult for a mother to understand healthy emotional reactions during the motherhood transition and this can worsen the degree of empathy and the relationship with the child, fueling the vicious circle of postpartum depression (13). Alternatively, many mothers are hesitant to disclose their illness out of fear of losing custody of their child (14). Lastly, cultural factors related to gender preference (e.g., preference for male children) and expectations around social behaviors of mothers often affect treatment-seeking behaviors and worsen stigma toward perinatal mental illness (13, 15).

## Interventions to reduce stigma toward perinatal mental illness

Perinatal women with mental illness require supportive care. Despite this, the majority of women are hesitant to discuss their illness with family members and health care workers (HCWs) due to stigma (16). The lack of knowledge about the spectrum of perinatal mental disorders not only increases the stigma associated with the illness, but also limits access to appropriate care for these conditions (6, 16). Although, Electroconvulsive therapy (ECT) is recommended as safe for women with clinical emergencies of perinatal mental disorders (e.g., catatonia, no food or fluid intake, suicide risk) (17). There is a scarcity of data from LMICs on ECT use and its effectiveness for perinatal disorders. ECT is often considered as the least helpful/harmful and inhumane treatment by the general public in LMICs (18). Both ECT and psychotropic medications are stigmatized as primary treatments for psychiatric conditions in pregnant women (6, 19, 20).

An intervention aimed at mitigating the mental illness stigma has been developed and evaluated for its effectiveness in LMICs. These interventions have been shown to be effective in enhancing the knowledge of healthcare professionals (HCWs) and the general community. However, they are minimally effective in improving the attitudinal and behavioral outcomes (21).

## Discussion

We found that there is a dearth of research pertaining to the epidemiology, impact, and interventions of mental illness stigma among perinatal women in LMICs. The determinants of mental illness stigma among perinatal women are more likely to differ from other groups of people. This is primarily due to prevailing myths and misinformation regarding the effect of psychotropic medications and ECT on mothers and child across the world including LMICs (6). Moreover, there is a dearth of targeted interventions that specifically address the perinatal mental illness stigma. The collaborative or integrative models [e.g., Brief Psychological intervention during pregnancy BIND-P model (22), Task sharing model (23), Stepped care model (24)] developed in LMICs with the goal of providing screening for common mental disorders (22, 25), and psycho-education have the potential to reduce stigma (inclusive of knowledge, attitude and behavior) and improve access to care for perinatal mental disorders (21). Thus, there is an urgent need to develop HCWs-based psychosocial interventions (including psychoeducation, and training) for stigma reduction (6, 26) (Table 1).

The cultural factors, such as collectivism, Confucianism, face concern and familism, religion, and supernatural beliefs, have a role in shaping stigmatizing behavior and attitudes toward perinatal mental health. Therefore, it is crucial to promote culture-specific mental health services and interventions for reducing stigma, which is a significant barrier to recovery. We used a basic theme-based approach to explore different types of stigma, assess their impact, and offer intervention. The approach has potential to guide for future research and collaboration, as well as the development of targeted theme-based interventions.

## The way forward

Due to significant disparities in mental health infrastructure across the world (27), a collaborative framework to address stigma toward perinatal mental illness across countries is required. Such framework should consider cultural, social, and health system-related factors while developing and adopting a psycho-social intervention for stigma toward perinatal mental illness. The existing infrastructure (e.g., community mental health model) of each country should be explored to develop such models of care or interventions. Further, there is an immediate need to undertake cross-country research to explore variations in stigma toward perinatal mental illness and develop tailored interventions to improve access to perinatal mental health services. These efforts have a potential to reduce the burden of perinatal mental health conditions and infant mental disorders across the world. There is a need for ECPs to work together and explore innovative methods to tackle the stigma associated with perinatal mental health in LMICs, such as digital strategies. The digital strategies can expand the reach of or complement the proposed interventions (Table 1). There are, however, several factors that need to be considered when proposing interventions and digital strategies: cultural adaptation to diverse contexts and settings, consideration of reliable measurement of stigma related to mental illness, and the risk that digital media could exacerbate stigma related to mental illness and spread misinformation.

## Conclusion

Stigma reduction programs that explicitly address perinatal mental disorders in LMICs are needed.

Effective intervention components, such as educational methods (knowledge, myth-busting), should be incorporated into these programs. Further, there is an urgent need to develop evidence based or culturally adapted interventions to reduce the stigma of perinatal mental disorders in LMICs. The implementation of evidence-based interventions aimed at reducing stigma and discrimination has the potential to improve help-seeking behavior and facilitate access to suitable mental health care in LMICs.

## Author contributions

AP: Conceptualization, Investigation, Writing—original draft. SP: Conceptualization, Investigation, Writing—original draft. MK: Conceptualization, Writing—original draft. SE: Writing—review & editing. RF: Writing—review & editing. RR: Conceptualization, Funding acquisition, Supervision, Writing—original draft, Writing—review & editing. MH: Writing—review & editing. MO: Writing—review & editing. MS: Conceptualization, Supervision, Writing—review & editing.
